# Clinical efficacy and therapeutic value of delayed surgery in patients with symptomatic old thoracolumbar fractures

**DOI:** 10.1186/s12893-021-01240-0

**Published:** 2021-06-11

**Authors:** Pan Li, Yunfei Huang, Zhuowen Liang, Lu Gan, Bin Wei, Zhengxu Ye, Mo Li, Zhuojing Luo

**Affiliations:** 1 Medical Research Institute, Northwestern Polytechnical University, Xi’an, China; 2 Department of Orthopaedics, Xijing Hospital, Air Force Medical University, 127 West Changle Road, Xi’an, 710032 China; 3 Department of Spine Sugery, Xi’an Jiaotong University Affliated Honghui Hospital, Xi’an, China

**Keywords:** Symptomatic old thoracolumbar fractures, Operative treatment, Neurologic function, Clinical efficacy, Treatment value

## Abstract

**Background:**

To investigate the clinical efficacy and therapeutic value of posterior decompression reduction, bone grafting fusion, and internal fixation for treatment of symptomatic old thoracolumbar fractures.

**Method:**

Retrospective analysis was conducted for 14 patients (9 men, 5 women; average age 40.1 years) with old thoracolumbar fractures who underwent posterior operation. American Spinal Injury Association (ASIA) scores were used to evaluate neurologic function. Vertebral body height, Cobb angle in the sagittal plane, spinal canal volume ratio (%) and bone graft fusion were analyzed by radiography and computed tomography on different follow-up times.

**Results:**

Mean follow-up was 27.1 months (23–36 months). Of three patients with ASIA grade A, 2 had improved postoperative urination and defecation, although no classification change. Preoperative ASIA score for eight patients with incomplete injury was grade B; four patients recovered to grade C at final follow-up. Preoperative ASIA score was C in three patients, increased to D in two patients and returned to normal E in one patient. Preoperative results showed average injured vertebra height loss rate decreased from 50.4 to 8.9%; average Cobb angle on the sagittal plane recovered from 39.6 to 6.9°; and the average spinal canal volume ratio recovered from 33.8 to 5.9%. Bony fusion was achieved; local lumbago and leg pain were relieved to some extent. No patients exhibited loosening of the fracture treated by internal fixation, pseudoarthrosis, or other related serious complications.

**Conclusion:**

Treatment of old thoracolumbar fractures by posterior decompression reduction, bone grafting fusion, and internal fixation can relieve spinal cord compression, improve neurologic function of some patients (ASIA grades B–C), effectively relieve pain, correct deformity, restore biomechanical stability, and significantly improve quality of life.

## Introduction

Thoracolumbar fracture is the most common type of spinal fracture caused by high-energy trauma [1], with most of these fractures occurring at the junctional area where the mechanical load is maximal [2, 3]. Because 40–80% of thoracolumbar spine injuries occur in a high-energy setting, such as motor vehicle accidents and falls, about one-third of patients with spinal cord injury have varying degrees of neurologic deficits, often accompanied by multiple organ damage [4]. In theory, thoracolumbar fractures should be treated with one-stage operation. However, because of life-threatening comorbidity such as brain injury, acute respiratory distress syndrome (ARDS), and abdominal trauma, the patients with severe thoracic fracture are unable to undergo early surgical treatment, or if they are treated with improper surgical procedures, such as poor surgical reduction, unreliable fixation, and bone graft fusion without fixation after laminectomy, they may experience poor outcome with delayed recovering course, delayed union or non-union thoracolumbar fracture [5].

With disease progression, thoracolumbar fracture develops into kyphosis, leading to further deterioration of neurologic function. According to the Spine Society of Europe, fractures more than 1 month after injury are considered old fractures [6]. Significant controversy is ongoing regarding the surgical effect and value of old thoracolumbar fractures [7]. Because of the difficulty and trauma of surgery and the potential for forced surgical reduction to easily damage large blood vessels and thoracic organs [8], create high hospitalization costs [9], and result in poor recovery of neurologic function after surgery [10], many patients with old thoracolumbar fractures have not been effectively treated. However, in the face of old fractures, surgical treatment is still recommended to avoid further compression of the fractured fragments, aggravating kyphosis, and damaging to neurological function [11]. Through surgical treatment, patients are encouraged to do mobilization and return to work as soon as possible. The latest report on symptomatic old osteoporotic vertebral compression fractures indicate that surgery could better treat this condition and restore spinal stability [12]. The current literature is more focused on elderly osteoporotic thoracolumbar fractures, there are fewer articles about symptomatic old fractures.

This retrospective clinical study assessed 14 patients with severe old thoracolumbar fractures. According to the classification standard revised by the American Society for Orthopedic Surgery (AO) spinal cord injury classification, all cases were anterior and posterior element injuries with distraction (Type B) or rotation (Type C). The purpose of this study was to explore the clinical efficacy and value of posterior decompression reduction, bone grafting fusion, and internal fixation for old thoracolumbar fractures and to observe whether decompression and structural reconstruction of old thoracolumbar fracture can improve the neurologic function and quality of life for patients.

## Materials and methods

### Patient population

From June 2014 to June 2019, 30 patients with old thoracolumbar fractures were identified in this study. A total of 14 patients met the inclusion/exclusion criteria: 9 men and 5 women age 21–61 years, with an average age of 40.1 years. Causes of injury were as follows: high fall for two patients, traffic-related impact for 7, bruising for 3, and coal mine explosions for 2. Damaged segments of the thoracic (T) and lumbar (L) vertebrae were as follows: T10–T11 for four patients, T11–T12 for 4, T12–L1for 2, L1 for 1 and L1–L2 for 3. American Spinal Injury Association (ASIA) classifications of spinal cord injury were as follows: grade A for three patients, grade B for 8, and grade C for 3. The time from injury to surgery was 1.5–6 months, with an average of 3.5 ± 0.9 months. All patients were examined by radiography, computed tomography (CT), and magnetic resonance imaging (MRI) before operation. The kyphotic angle was measured on lateral radiography in terms of angles between the upper surface of the first normal vertebra above the lesion and the lower surface of the first normal vertebra below the lesion [13], and the Cobb angle was 30.5– 46.1°, with an average of 39.6 ± 4.1°. The preoperative average height loss rate was 50.4% and the average preoperative volume ratio of the spinal canal was 33.8%. All 14 patients and their families were informed of the study and signed informed consent forms.

### Inclusion criteria

Inclusion criteria were as follows: (1) radiography, CT, magnetic resonance imaging confirmed old fractures and sagittal Cobb angle ≥ 30°; (2) strict nonsurgical treatment was ineffective for severe low back pain combined with neurologic dysfunction; and (3) fractures and dislocations only involved 2 or fewer vertebrae.

### Exclusion criteria

Exclusion criteria were as follows: (1) kyphosis caused by tuberculosis or tumor; (2) inability to tolerate surgery because of other serious diseases, such as severe cardiac, hepatic, and renal insufficiency; and (3) blood, immune, and mental health diseases.

### Surgical technique

Under general anesthesia, the patients were placed in a prone position, and vital signs were monitored during the operation. The surgical team chose a posterior median incision to cut the skin, subcutaneous tissue, and deep fascia; exposed the fractured vertebral body and its two upper and lower segments; and exposed the outer edge of the facet joint. In the process of stripping the paravertebral muscle, the pressure of elevator was strictly controlled to prevent further spinal cord injury caused by the fracture of the lamina into the spinal canal.

For patients with visible nerve fiber compression, the lamina was retracted with a 2 mm lamina bone bite forceps in the normal lamina space, and the nerve fibers were carefully released to avoid the secondary injury of the spinal cord. The nerve fibers were carefully placed into the dural sac after repair. Pedicle screws were inserted into the upper and lower segments of the injury segment. The spinal cord was carefully pulled with a nerve hook, and the corresponding nerve roots were protected. After reaching the intervertebral space, the upper vertebral cartilage endplate was carefully scraped with a reamer and a scraper to expose the bony endplate.

For burst vertebral fractures and anterior vertebral wall, it was essential to pay attention to avoid accidental injury of anterior vertebral vessels. For patients with different pathologic features and degrees of kyphosis, different osteotomy methods were selected for spinal shortening and screw rod system internal fixation. If the reduction of severe fracture was difficult, then the periosteal stripping device was inserted into the bilateral articular process of dislocation space to attempt restoration so that the upper and lower spinous processes are aligned in the sagittal and coronal planes. After a satisfactory reduction was confirmed with the intraoperative C-arm monitor, the pedicle screw could be inserted into the vertebral body under direct vision. Then the SF bracket was placed and pressed to reduce the distance between the two fractured vertebraes and to complete the orthopedic procedure. Interbody spinal fusion with bone particles was trimmed from the spinous process, lamina, and bone fragments of vertebral body. After washing with normal saline, the spinal cord and nerve root were explored again, the herniated spinal cord fibers were retrieved, and the nerve roots were repaired or displaced. The wound was closed layer by layer after placing the drainage tube. The operation duration, intraoperative blood loss, and neuroelectrophysiologic monitoring results were recorded.

### Postoperative care

All patients were routinely treated with antibiotics 24 h after surgery to prevent postoperative infection. The drainage tube was retained for 48–72 h postoperatively, and indwelling catheter was placed. The patients were asked to actively or passively move the limbs to prevent deep vein thrombosis and muscle atrophy. After routine bed rest for 8–12 weeks, patients began to exercise with a walking aid. Patients who could not yet walk choose to perform wheelchair exercise.

### Follow-up evaluation

Patients underwent radiography on postoperative Day 2 to evaluate the degree of decompression and the placement of bone graft and internal fixation. Subsequent follow-up evaluation was at 3, 6, and 12 months postoperatively and annually thereafter. The related complications were observed. Radiography and CT were used to evaluate the recovery of sagittal reduction, decompression, fusion, loss rate of injured vertebral height, Cobb angle correction, and spinal canal occupancy rate. At each follow-up evaluation, radiographic studies obtained from standing and dynamic flexion–extension positions were used to determine fusion status, development or progression of postoperative deformity, and failure of internal fixation. For cases in which the fusion state was indefinite on radiographically, CT scans were performed. Clinical and radiological definition of successful fusion were as follows: no local pain and tenderness, no motor abnormalities, no correction loss, and internal fixation failure. Bone fusion was identified by formation of trabecular bony bridges between contiguous vertebral bodies through CT scans [14].

### Statistical analysis

A paired *t* test was used to compare the height loss rate of the vertebral body, the Cobb angle on the sagittal plane, the volume ratio of the spinal canal preoperatively, postoperatively, and at the last follow-up. Statistical analysis was conducted using SPSS for Windows (version 23.0; IBM, Armonk, NY, USA), with *p* < 0.05 considered statistically significant.

## Results

The same group of chief physicians led the operation of 14 patients with old thoracolumbar fractures. Table 1 summarizes the patient data. Average follow-up was 27.1 months (range, 23–36 months). No one died of postoperative complications. One case of pain still exists, one case of urinary tract infection.

**Table 1:** Summary of Clinical Data for 14 Patients With Old Thoracolumbar Fractures

Case no	Age(y)/sex	Mechanisms of injury	Level	Duration of disease (we)	Neurologic status(ASIA)		The vertebral height loss rate(%)		Cobb angle (°)		Volume ratio of the spinal canal (%)		Follow-up (mo)	Postoperative complication
					Pre	FFU	Pre	FFU	Pre	FFU	Pre	FFU		
1	41, F	F	T10-T11	5	A	A	41.2	8.1	42.6	8.7	37.6	7.1	23	
2	28, M	TA	T11-T12	6	C	E	46.5	5.7	38.7	5.9	35.4	6.8	24	
3	21, F	TA	T11-T12	8	A	A	47.4	8.4	39.9	8.3	36.4	5.4	25	
4	45, M	TA	T11-T12	5	C	D	53.5	8.9	42.8	6.3	34.6	4.5	24	
5	59, M	BI	T10-T11	24	B	B	41.1	7.9	45.4	5.2	33.2	7.0	24	Urinary tract infection
6	29, M	BI	T11-T12	23	B	C	34.5	5.9	46.1	7.6	31.8	5.9	23	
7	43, F	TA	T12-L1	18	B	B	40.6	11.5	38.3	5.6	31.8	6.5	25	
8	45, M	BI	T12-L1	21	C	D	68.5	10.9	38.3	6.2	31.2	4.9	34	
9	25, M	ME	L1-L2	9	B	C	49.2	9.1	38.7	7.5	30.0	6.1	36	
10	32, M	TA	T10-T11	23	A	A	37.4	8.3	39.4	6.3	37.0	6.4	25	
11	61, F	F	L1	10	B	C	88.1	11.2	30.5	7.5	31.5	3.0	29	
12	27, M	TA	L1-L2	8	B	C	49.4	8.9	40.5	6.8	34.5	5.6	28	
13	56, M	ME	T10-T11	9	B	B	45.3	8.9	35.7	7.9	35.3	6.5	31	Persistent pain
14	49, F	TA	L1-L2	12	B	B	62.3	10.3	37.5	6.9	32.7	6.3	29	

*Pre* preoperative, *FFU* final follow-up, *mo* month, *we* week, *F* fall, *TA* traffic accident, *ME* mine explosion, *BI* bruise injury, *T* thoracic vertebra, *L* lumbar vertebra

### Operation time and bleeding volume

Mean operation time was 243 min (range, 120–335 min), and mean bleeding volume was 525 mL (range, 410–2100 mL).

### Radiologic evaluation

**Fig. 1:**
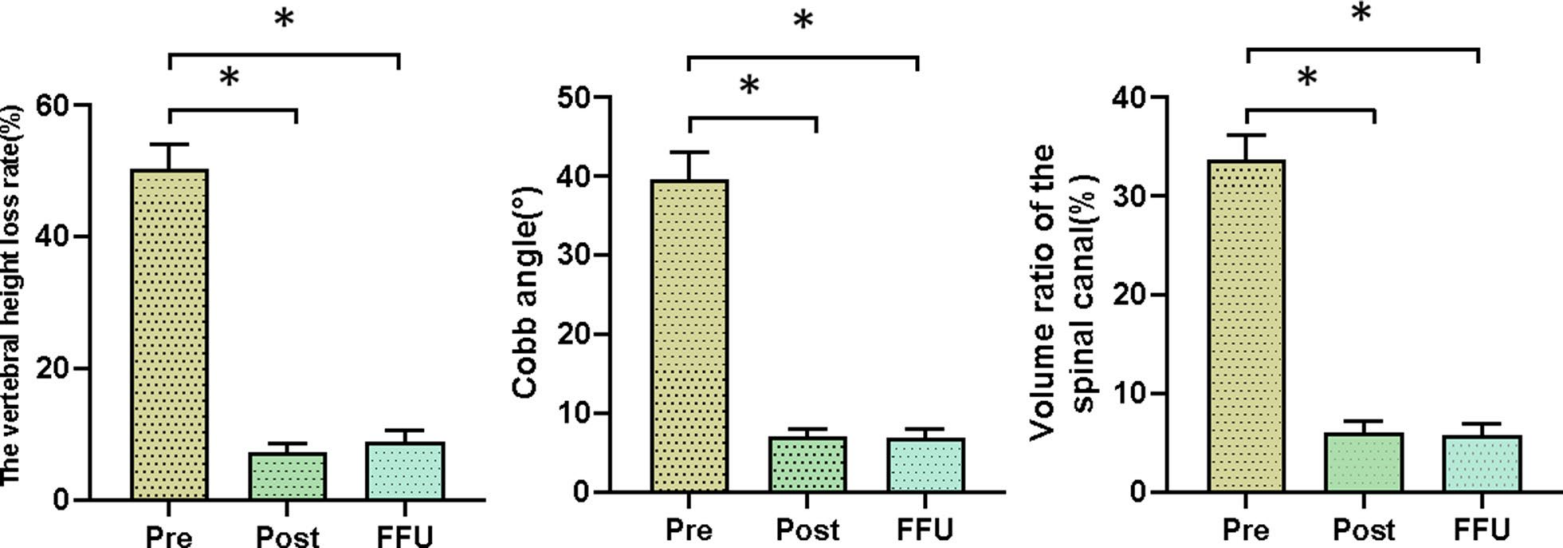
Changes of the vertebral height loss rate, Cobb angle, volume ratio of the spinal canal at different times. *Pre* preoperative, *Post* postoperative, *FFU* final follow-up. Compared with preoperative, *P < 0.05

All patients showed successful bone fusion, no significant loss of vertebral height and intervertebral height, and a good position of the pedicle screw. The preoperative average height loss decreased from 50.4% (range, 34.5%–88.1%) to 7.2% (range, 4.7%–9.5%) postoperatively *(p* < 0.05 vs. preoperative) and to 8.9% (range, 5.7%–11.2%) at the final follow-up (*p* < 0.05 vs. preoperative). The average Cobb angle on the sagittal plane was corrected from 39.6° (range, 30.5°–46.1°) preoperatively to 5.7°(range, 5°–8°) postoperatively (*p* < 0.05 vs. preoperative) and 6.9°(range, 5.2°–8.7°) at the final visit (*p* < 0.05 vs. preoperative). The average preoperative volume ratio of the spinal canal recovered from 33.8% (range, 30%–37.6°) to 6.4% (range, 4%–8%) postoperatively (*p* < 0.05 vs. preoperative) and to 5.9% (range, 3–7.1%) at the final follow-up (*p* < 0.05 vs. preoperative). No significant loss of deformity correction was noted in these patients. Figure 1 showed the changes of the vertebral height loss rate, Cobb angle, volume ratio of the spinal canal at different times.

### Clinical outcomes

**Fig. 2:**
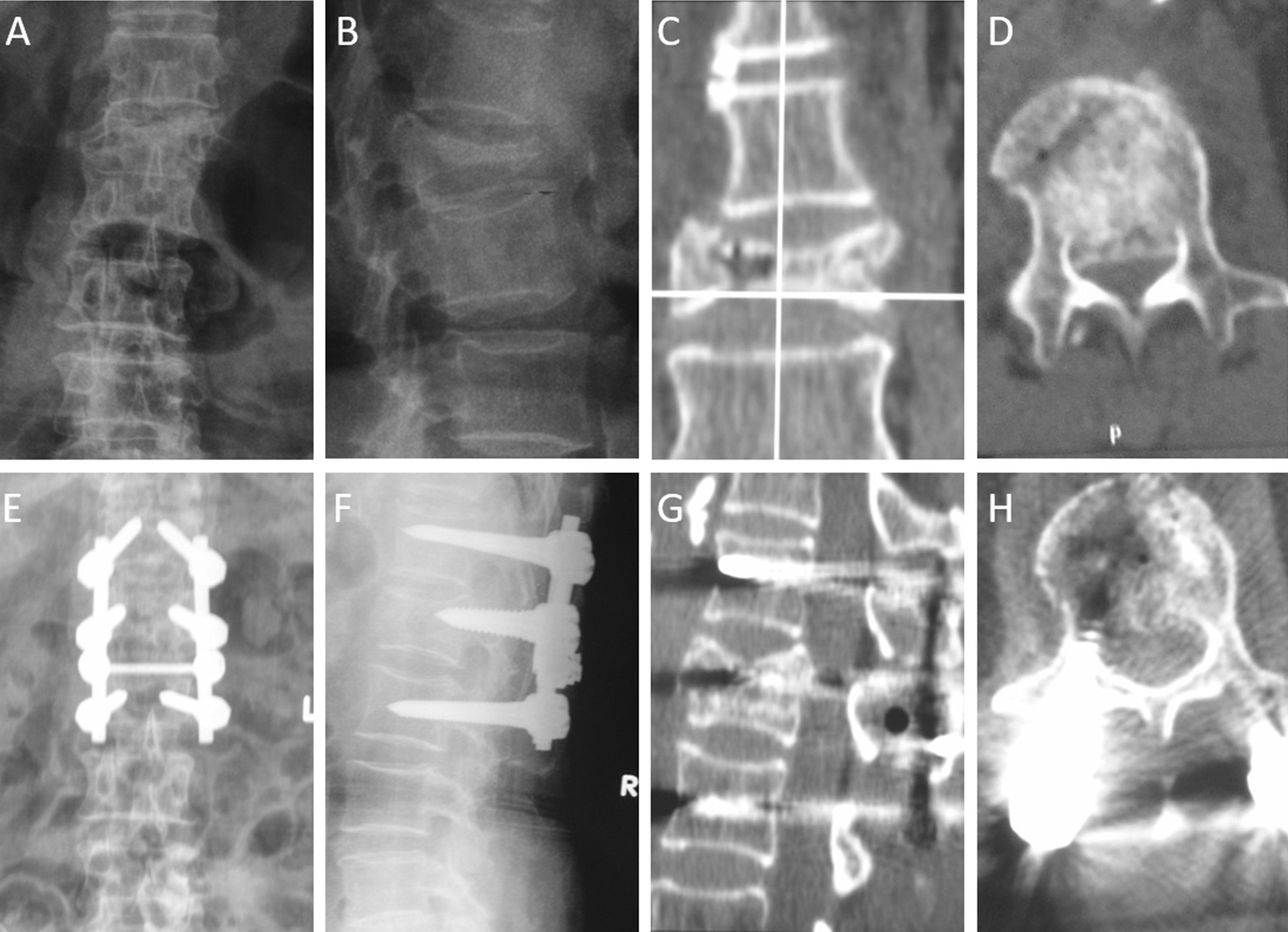
A 61-year-old female patient with back pain, AO type B burst fracture of Lumbar 1, and ASIA grade B paralysis due to fall injury. The preoperative anteroposterior X-ray view **a** and lateral X-ray view **b** showed the collapsed, burst and wedge-shaped L1 vertebra. The interpedicular distance was widened and the pedicle was deformed. The preoperative coronal CT **c** and axial CT **d** showed the decreased height of the vertebral body, the longitudinal fissure, vacuum phenomenon, and spinal canal space occupyed by burst fracture fragment. The 2nd day postoperative anteroposterior **e** and lateral X-ray **f** view showed a good position of the instrumentation, well reduced L1 vertebra with good sagittal alignment and height. The Sagittal CT image **g** showed the height of the vertebral body has been expanded and restored. Axial CT image **h** showed fracture fragment was reduced with good restore of the spinal canal

Based on the ASIA grading system, the neurologic deficits for some patients were improved at the final follow-up. No change in ASIA grade was observed for bilateral lower limb paralysis in three patients with grade A, but two patients had improved postoperative urination and defecation. The preoperative ASIA score for eight patients with incomplete injury was grade B, and four patients recovered to grade C at the final follow-up. The preoperative ASIA score was grade C for three patients, which increased to grade D in 2 patients and returned to grade E in 1 patient. Typical patients are shown in Fig. 2.

## Discussion

Patients with thoracolumbar fractures, especially those with burst fractures, may experience disease progression to kyphosis if they are not treated promptly. The deformity causes the gravity line of the spine to move forward, which leads to the extension of the resistance arm to maintain the balance and stability of the spine. The anterior and middle columns of the spine bear excessive compressive stress and the columns bear excessive tensile stress. The long-term centrifugal load on the spine aggravates the kyphosis. For these patients who gradually developed into old thoracolumbar fractures, residual fragments in the spinal canal continue to compress nerves or cause spinal instability. In general, spinal deformity, spinal instability, or increased neurologic deficits are surgical indications [15]. Posterior spinal surgery can effectively avoid blood vessels and important organs. This surgery has the advantages of being a low-risk, relatively simple operation with accurate short-term effects [16], which has led it to be one of the most commonly used surgical approaches for the treatment of old thoracolumbar fractures. With the improvement of posterior surgery technology, surgeons have a new understanding of one-stage posterior surgery and the potential improvements.

Other studies of thoracolumbar fractures have used different approaches. Machino [17] used transforaminal thoracic interbody fusion in the treatment of lower thoracic spine fracture dislocations, which achieved posterior rigid fixation with instrumentation and anterior column reconstruction by interbody fusion in 7 patients. Hao [18] used the intervertebral foramen approach in the treatment of thoracolumbar fracture dislocation, which showed good results at 2 year follow-up. These previous studies primarily addressed new spinal fractures, whereas in the current study the 14 patients selected had severe old fractures with neurologic impairment. Therefore, this study intended to explore the clinical efficacy and value of posterior decompression reduction, bone grafting fusion, and internal fixation for old thoracolumbar fractures and to observe whether decompression and structural reconstruction of thoracolumbar old fracture could improve the neurologic function and quality of life of patients.

Surgical treatment for severe old thoracolumbar fractures is difficult to perform because the dislocation of the vertebral body results in severe stranded facet joints and because of the presence of hematoma and scar adhesions around the fracture vertebral body, a cauda equina nerve tear, and injury of large blood vessels on the ventral side of the vertebral body [19]. Therefore, the preoperative evaluation of abdominal organ injury is particularly important [20]. It is necessary to treat important organs first, and orthopedic surgery can be carried out after the treatment of substantive organs stabilizes the patient. According to the Advanced Trauma Life Support(ATLS), injures that impair respiratory and circulatory function have to be treated with priority. Assessment of spinal injuries is usually performed secondarily [21]. All 14 patients in this group underwent surgery with stable vital signs. The main difficulties and precautions for this operation are as follows:

- When exposing the surrounding area of the injured vertebra, because of the changes in anatomic structure, especially fracture dislocation, attention should be paid to avoiding damage to important structures such as dura and nerve root.
- Patients with severe fracture and dislocation often have different degrees of damage to the pedicle; therefore, it is better to insert the pedicel screw using the navigation perspective when operating. In principle, the range of fixation is the upper and lower segments of the injured vertebra, and whether the screw is placed on the injured vertebra depends on the degree of pedicle injury.
- Attention should be paid to the intensity of surgical traction reduction. First, the ratchet forceps can be used to clamp the lifting reduction, and the reduction or partial reduction can be achieved by combining the horizontal position and axial traction. The traction with excessive force may tear the aorta and inferior vena cava that have been tightly adhered to the anterior vertebral body. If the posterior reduction is difficult, then the periosteal stripping device can be used to place bilateral articular processes of the dislocation vertebral body for poking reset. For old fractures, it is critical to avoid blindly pursuing complete reduction because this approach may damage the aorta and increase the risk of spinal cord injury.
- For patients with severe kyphosis requiring osteotomy, the articular surface must be excised and the soft tissues of transverse process, lamina, and spinous process outside the facet joint removed. The upper and lower lamina must be completely bitten, grinded to bleeding, and the cancellous bone fragments mixed with artificial bone materials to implant the transverse process space, lamina, and articular process for fusion to ensure the close alignment.
- For patients with old thoracolumbar fractures, after posterior open reduction, if the bone trabecula and nucleus pulposus structure of the injured vertebral body did not completely reset, then the “empty vertebral body” phenomenon may occur or the surgery may not achieve the purpose of open reduction such that the anterior and middle columns lose the integrity of the structure. If these patients were not given effective bone grafting, internal fixation fatigue, fracture, collapse of fractured vertebral body and loss of correction can occur in the late stage. In this regard, the authors of this current study perform vertebral bone grafting through pedicle at the same time combined with posterior intertransverse process bone grafting to achieve spinal stability. The authors’ surgical purpose is to provide the best conditions for spinal cord recovery.

Analysis of the statistical results for follow-up of the 14 study cases showed the postoperative sagittal Cobb angle and thoracolumbar sequence improved satisfactorily. During the follow-up period, no further loss of vertebral height and force line occurred, and different degrees of neurologic recovery were observed at final follow-up. In addition, 2 patients with ASIA grade A experienced improvement in urination and defecation, and 4 patients with ASIA grade B advanced to grade C. Among the 3 patients with ASIA grade C, 2 patients were improved to grade D, and 1 patient was restored to grade E. The patients’ back pain was relieved to some extent postoperatively. Previous studies have shown that operation has some complications, such as pseudoarthrosis, epidural hematoma, infection and so on. Urinary tract infection (UTI)occurred in one of 14 patients, he received timely antibiotic treatment. UTI represent a common perioperative complication among elderly patients. For this kind of complication, early detection of symptomatic UTI and targeted antibiotic treatment are very important, perioperative care is also critical to prevent prolonged clinical courses [22]. One patient still had persistent pain after operation. However, the causes of pain are controversial, which may be the severe preoperation pain, lumbar instability, depression, nerve stimulation and so on. Reduction of excessive traction of spinal cord during operation and bone graft fusion in fixed area or injured segment are effective measures to reduce delayed pain, nerve root symtoms, loss of correction degree and other complications. The existing data in the literature show a contradictory view of the surgical treatment of thoracolumbar fractures. The surgical supporters believed that the removal of compressed fracture blocks is conducive to the recovery of neurologic function, preventing further deterioration, and reducing kyphosis, whereas the opponents proposed that the removal of compressed fragments of spinal cord did not represent the recovery of neurologic function. However, studies have reported that surgical treatment is superior to conservative treatment in neurologic function recovery [23]. Conservative treatment of patients with spinal cord injury may have a risk of up to 10% neurologic deterioration [24]. According to the guidelines, surgical treatment is recommended when the Thoracolumbar Injury Classification and Severity Scale (TLICS) score is higher than 4 points, whereas conservative treatment is recommended when the TLICS score is lower than 4. When the TLICS score is equal to 4 points, the appropriate scheme should be selected according to the patient’s condition [25, 26]. A double-blind clinical trial conducted by Alireza Mohamadi found that for cases in which the TLICS score was equal to 4 points, based on the local sagittal angle and regional sagittal angle and visual analog scale score, the recovery effect of the surgery group was better than that for the conservative treatment group [27]. These researchers attributed this difference to the method of execution. In a prospective cohort study at the Orthopedic Trauma Center of Western China by Du’s research team, patients with AO type B and type C should undergo surgery as early as possible to achieve better clinical results [28]. In addition, in 2011, Fang first reported a case of transforaminal lumbar interbody fusion for which surgery was performed in the treatment of an old T12/L1 fracture [19]. Results showed that the clinical effect was good, suggesting that transforaminal lumbar interbody fusion may be a treatment for old thoracolumbar fractures.

At present, orthopedic surgeons generally believe that spinal canal decompression can improve neurologic function in patients with thoracolumbar burst fractures [29]. Scholars have proposed their own surgical indications, such as spinal canal occupancy rate of 25%, 50%, or more. The experiences of the authors of the present study are as follows based on the degree of kyphosis. For kyphosis caused by thoracolumbar fractures, which leads to local symptoms and neurologic symptoms, and when conservative treatment cannot solve low back pain and neurologic symptoms, radiographic hyperflexion and hyperextension showed that vertebral slip > 5 mm or local sagittal rotation > 15° can be identified as chronic instability, and surgery is needed. These cases also require surgery: a kyphosis angle is ≥ 30°; the presence of lumbar muscle strain caused by kyphosis, or secondary symptoms of intervertebral osteoarthritis and neurologic symptoms that cannot be alleviated. According to the injury of vertebral body, the commonly used osteotomy approaches include Smith-Petersen osteotomy, pedicle subtraction osteotomy, posterior vertebrae column resection, and vertebral column decancellation [30]. The goal is to restore the curvature, and then reliable bone graft is needed in the osteotomy area to increase fusion.

For patients with insignificant improvements in postoperative symptoms, however (such as the 3 patients with ASIA grade A and 4 patients with B in this group who did not improve), the necessity of surgery must be analyzed. Some in the field think there is no need for surgery for patients with severe trauma and hopeless neurologic recovery. However, some scholars believe that for patients with old thoracolumbar fractures and spinal cord compression, spinal decompression surgery can improve the local blood supply and metabolism of the spinal cord, which is conducive to nerve rehabilitation, and that surgery can significantly reduce preoperative refractory pain and improve defecation function [2], even if it cannot significantly improve the sensory and motor function of the lower limbs. Trafton [31] believed that T12 or L1 burst fractures can be operated if the sagittal diameter of the spinal canal is reduced to 50% or more. The recovery of spinal cord nerve is slow and closely related to the degree of spinal cord injury of the patient, which is affected by many factors, such as ASIA grade before injury [32], operation time [33], age, injury segment, sex, health status, and postoperative rehabilitation training. In general, 5%–10% of patients with complete spinal cord injury have improved to some extent [34], which is consistent with the results of this current study. In addition, Vaccaro [35] indicated that the overall outcome after surgical treatment for posttraumatic malformations is satisfactory and that early treatment is more effective than late treatment. In the current study, for 4 patients younger than 30 years, neurologic function recovered well, and ASIA grade increased by 1 or 2 grades. Among these patients were 2 men age 25 and 27 years who experienced severe thoracolumbar fracture caused by an injury from a coal mine blast and a car accident, respectively, and they both recovered well after surgery. Therefore, the authors of this current study believe that young patients are more likely to obtain the recovery of neurologic function. The spinal cord is mixed with the cytokines of the lower motor neurons of the spinal cord cone and the axons of the lower motor neurons in the cauda equina, and these structures have complex pathophysiological processes and different potential for neurologic recovery after injury. In contrast, poor nervous system effects and prognosis have been reported for older people [36]. Sewell [37] also pointed out that neurologic recovery is more likely in young patients. In spinal cord injury, it is more difficult to restore neurologic function for patients with thoracic injury than for patients with cervical injury. In anatomy, the lower lumbar nerve has reached the root, and it has good pressure resistance and traction resistance, which is stronger than that of spinal cord nerve. Good therapeutic effect can still be obtained after decompression surgery. Studies have shown that if the injured segment is close to the lower lumbar spine, the postoperative recovery will be better because the spinal cord is absent from these vertebrae. For the current study however, due to the small sample size and short follow-up time, no statistical difference was noted in the relevant data for nerve recovery in this group.

In China, severe thoracolumbar fractures often occur in low-income groups, i.e. injuries caused due to mining work and falls from high altitudes in construction laborers. Often in these patients, the injuries are serious, the clinical recovery is unclear, and high medical expenses are required to be borne. Especially in patients with old fractures, if there is little or no improvement in neurologic function after incurring high medical expenses, further attempts at treatment may cause medical disputes. Therefore, the current authors conducted a preliminary study on whether the surgical treatment of old fractures has a positive effect on the improvement of neurologic function. All the cases collected in this study were severe thoracolumbar fractures, yielding a small number of cases (14 cases) and a short follow-up time (average 27.1 months). The long-term effect of postoperative recovery in these patients is not clear. Although the short-term effect of some patients is ideal, multicenter, large sample, and longer-term follow-up results are still needed to verify the clinical efficacy of this operation.

## Conclusion

In summary, through this analysis the authors believe that the short-term clinical curative effect for patients with symptomatic old thoracolumbar fractures is meaningful, and the treatment of preoperative injuries is the premise of good curative effect. These results preliminarily confirmed that posterior decompression reduction, bone grafting fusion, and internal fixation for old thoracolumbar fractures can not only relieve pain and correct deformity, but also improve the neurologic function for some patients (ASIA grades B–C), whereas the improvement of neurologic function of patients with ASIA grade A is limited.

## Data Availability

The datasets used and/or analysed during the current study available from the corresponding author on reasonable request.

## Abbreviations

ASIA: American Spinal Injury Association.
UTI: Urinary tract infection
